# *N*-(2-Chloro­eth­yl)morpholine-4-carbox­amide

**DOI:** 10.1107/S1600536814005832

**Published:** 2014-03-22

**Authors:** Oguejiofo T. Ujam, Jonnie N. Asegbeloyin, Brian K. Nicholson, Pius O. Ukoha, Nkechi N. Ukwueze

**Affiliations:** a Department of Pure and Industrial Chemistry, University of Nigeria, Nsukka, Enugu State, Nigeria; b Department of Chemistry, University of Waikato, Private Bag 3105, Hamilton, New Zealand

## Abstract

The title compound, C_7_H_13_ClN_2_O_2_, synthesized by the reaction of 2-chloro­ethyl iso­cyanate and morpholine, crystallizes with four molecules in the asymmetric unit, which have similar conformations and comprise two pairs each related by approximate non-crystallographic inversion centres. Two of them have a modest orientational disorder of the 2-chloro­ethyl fragments [occupancy ratio of 0.778 (4):0.222 (4)]. In the crystal, mol­ecules are linked by N—H⋯O=C hydrogen bonds, forming three crystallographically different kinds of infinite hydrogen-bonded chains extending along [001].

## Related literature   

For the solution-phase preparation of substituted morpholine derivatives, see: Lainton *et al.* (2003 ▶). For a related thio­morpholine analogue, see: Ujam *et al.* (2010 ▶); Henderson *et al.* (2006 ▶).
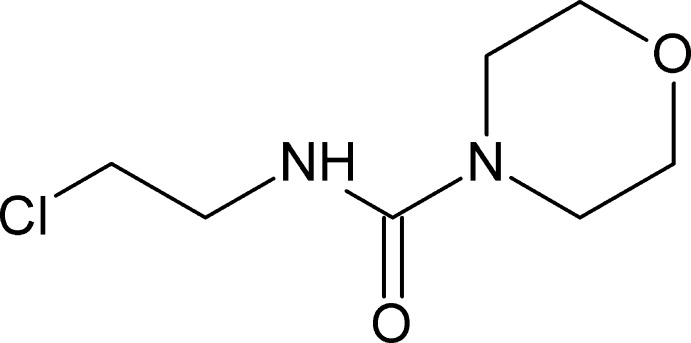


## Experimental   

### 

#### Crystal data  

C_7_H_13_ClN_2_O_2_
*M* *_r_* = 192.64
Monoclinic, 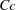

*a* = 10.7393 (8) Å
*b* = 33.613 (3) Å
*c* = 9.9942 (7) Å
β = 95.704 (5)°
*V* = 3589.9 (5) Å^3^
*Z* = 16
Mo *K*α radiation
μ = 0.39 mm^−1^
*T* = 99 K
0.30 × 0.10 × 0.10 mm

#### Data collection  

Siemens SMART CCD diffractometer
Absorption correction: multi-scan (*SADABS*; Sheldrick 2003 ▶) *T*_min_ = 0.892, *T*_max_ = 0.962
39446 measured reflections
8451 independent reflections
5276 reflections with *I* > 2σ(*I*)
*R*_int_ = 0.082

#### Refinement  

*R*[*F* ^2^ > 2σ(*F* ^2^)] = 0.059
*wR*(*F* ^2^) = 0.158
*S* = 1.04
8451 reflections
307 parameters
164 restraints
H-atom parameters constrained
Δρ_max_ = 0.56 e Å^−3^
Δρ_min_ = −0.59 e Å^−3^
Absolute structure: Flack (1983 ▶), 4226 Friedel pairs
Absolute structure parameter: 0.38 (17)

### 

Data collection: *SMART* (Bruker, 2001 ▶); cell refinement: *SAINT* (Bruker, 2001 ▶); data reduction: *SAINT*; program(s) used to solve structure: *SHELXS97* (Sheldrick, 2008 ▶); program(s) used to refine structure: *SHELXL97* (Sheldrick, 2008 ▶); molecular graphics: *ORTEP-3 for Windows* (Farrugia, 2012 ▶) and *Mercury* (Macrae *et al.*, 2006 ▶); software used to prepare material for publication: *WinGX* (Farrugia, 2012 ▶).

## Supplementary Material

Crystal structure: contains datablock(s) I. DOI: 10.1107/S1600536814005832/qk2064sup1.cif

Structure factors: contains datablock(s) I. DOI: 10.1107/S1600536814005832/qk2064Isup2.hkl

Supporting information file. DOI: 10.1107/S1600536814005832/qk2064Isup3.mol

Supporting information file. DOI: 10.1107/S1600536814005832/qk2064Isup4.cml

CCDC reference: 991972

Additional supporting information:  crystallographic information; 3D view; checkCIF report

## Figures and Tables

**Table 1:** Hydrogen-bond geometry (Å, °)

*D*—H⋯*A*	*D*—H	H⋯*A*	*D*⋯*A*	*D*—H⋯*A*
N2—H2*C*⋯O6	0.85	2.03	2.831 (6)	157
N4—H4*C*⋯O4^i^	0.86	2.00	2.826 (6)	162
N6—H6*C*⋯O2^ii^	0.85	2.00	2.819 (6)	161
N8—H8*C*⋯O8^iii^	0.82	2.05	2.809 (6)	153

Symmetry codes: (i) 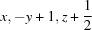; (ii) 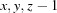; (iii) 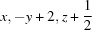.

**Fig. 1.**
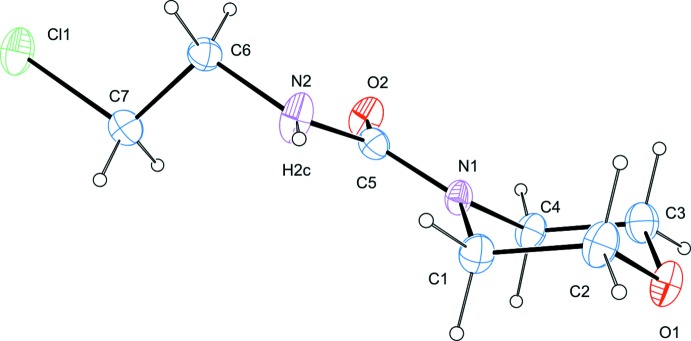
The molecular structure and atom numbering of one of the four independent molecules of the title compound with displacement parameters drawn at the 40% probability level for non-H atoms.

**Fig. 2.**
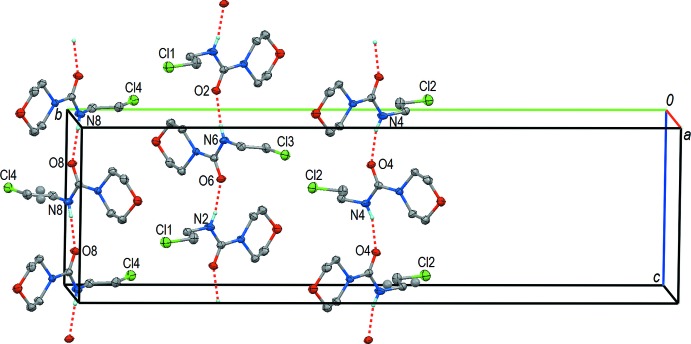
Crystal structure of the compound showing the four different intermolecular N—H···O═C hydrogen bonds (dotted lines) within the three different hydrogen bond chains along [001]. C-bonded H atoms omitted for clarity.

**Fig. 3.**
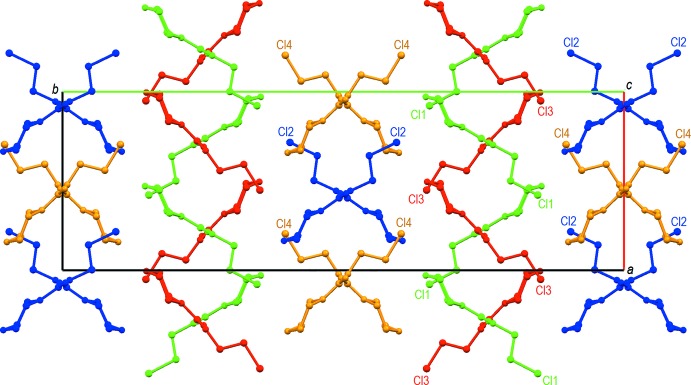
Packing diagram of the title compound viewed along [001], the hydrogen bond chain direction. Symmetry equivalent molecules are colour coded and only Cl atoms were labeled. H atoms omitted for clarity.

## supplementary crystallographic information

### 1. Comment

The title compound, N-(2-chloro­ethyl) morpholine-4-carboxamide, was prepared as part of an ongoing project investigating the multifunctional alkyl­ation of [Pt_2_(µ-S)_2_(PPh_3_)_4_] (Ujam *et al.*, 2010). The X-ray structure determination established the molecular structure and atom connectivity of the title compound C_7_H_13_Cl_1_N_2_O_2_ (Fig. 1). There are four independent molecules in the asymmetric unit which have the same overall conformation and comprise two pairs each related by approximate non-crystallographic inversion centres. Two of them have a modest orientation disorder of the 2-chloro­ethyl fragments. The molecules consist of a chair-shaped morpholine ring attached to a planar urea-type N_2_CO unit. The 2-chloro­ethyl side chains are oriented approximately perpendicular to the N_2_CO unit. In the crystal structure the molecules are linked by N—H···O═C hydrogen bonds (N···O = 2.809 (6) – 2.831 (6) Å) to form three crystallographically different kinds of infinite hydrogen bonded chains extending along [001] (Fig. 2), all with criss-cross patterns of molecule orientations when viewed along the chains (Fig. 3).

### 2. Experimental

Morpholine [HN(CH_2_CH_2_)_2_O, 200 mg, 0.002 mmol] was added to a solution of 2-chloro­ethyl iso­cyanate [ClCH_2_CH_2_NCO, 200 mg, 0.002 mmol] in di­ethyl ether (30 mL), immediately producing a white precipitate of the product. After stirring for 5 min the product was filtered and washed with ether (20 mL) and dried under vacuum to give ClCH_2_CH_2_NHC(O)N(CH_2_CH_2_)_2_O. Crystals suitable for X-ray crystallographic analysis were obtained by vapour diffusion of di­ethyl ether into a di­chloro­methane solution.

### 3. Refinement

Crystal data, data collection and structure refinement details are summarized in the crystallographic data Table. Diffraction images of the reciprocal space calculated from the recorded frame data were consistent with the crystallography reported and did reveal neither commensurate nor incommensurate superstructure reflections. All attempts to solve the structure in space group *C*2/*c* were unsuccessful, but it solved readily in *Cc*. The compound has four independent molecules in the asymmetric unit, comprising two pairs each related by an apparent inversion centre at *x,y,z* = 0.43, 0.63, 0.81 for the molecules 1 (Cl1, O1, O2, N1, N2, C1 through C7) and 2 (Cl2, N3, N4, O3, O4, C8 through C14), and at *x,y,z* = 0.42, 0.88, 0.31 for molecules molecules 3 (Cl3, O5, O6, N5, N6, C15 through C21) and 4 (Cl4, N7, N8, O7, O8, C22 through C28) . The two inversion centres were approximately related by 0 0.25 0.5 which is not a crystallographic relationship in *C*2/*c*, so the structure could not be converted to the centrosymmetric cell. Refinement was completed in the non-centrosymmetric space group *Cc*, and the crystal treated as a racemic twin. Unrestrained refinement led to a large spread in values for chemically equivalent bond parameters, and some unrealistic thermal ellipsoids, no doubt arising from instability associated with the pseudosymmetry. Hence the final refinement restrained the independent molecules to similar geometry using the SAME command of *SHELXL*, and EADP constraints were applied to equivalent atoms related by pseudosymmetry. Some residual electron density appeared to arise from partial disorder of the ethyl­ene groups of two of the molecules (molecules 2 and 4) over two sites (0.78:0.22), so the C atoms of the minor component were included with fixed isotropic thermal parameters. Otherwise all non-hydrogen atoms were treated anisotropically. H atoms attached to carbon atoms were included in calculated positions [*U*_iso_(H) = 1.2×*U*_equ_(C)], but those attached to the amide N atoms were refined with *DFIX* constraints and *U*_iso_(H) = 0.03 fixed.

### Crystal data

**Table:**

C_7_H_13_ClN_2_O_2_	*F*(000) = 1632
*M**_r_* = 192.64	*D*_x_ = 1.426 Mg m^−^^3^
Monoclinic, *C**c*	Mo *K*α radiation, λ = 0.71073 Å
*a* = 10.7393 (8) Å	Cell parameters from 3770 reflections
*b* = 33.613 (3) Å	θ = 2–27°
*c* = 9.9942 (7) Å	µ = 0.39 mm^−^^1^
β = 95.704 (5)°	*T* = 99 K
*V* = 3589.9 (5) Å^3^	Needle, colourless
*Z* = 16	0.30 × 0.10 × 0.10 mm

### Data collection

**Table:**

Siemens SMART CCD diffractometer	5276 reflections with *I* > 2σ(*I*)
Radiation source: fine-focus sealed tube	*R*_int_ = 0.082
ω scans	θ_max_ = 27.8°, θ_min_ = 1.2°
Absorption correction: multi-scan (*SADABS*; Sheldrick 2003)	*h* = −14→14
*T*_min_ = 0.892, *T*_max_ = 0.962	*k* = −43→44
39446 measured reflections	*l* = −13→13
8451 independent reflections	

### Refinement

**Table:**

Refinement on *F*^2^	Secondary atom site location: difference Fourier map
Least-squares matrix: full	Hydrogen site location: inferred from neighbouring sites
*R*[*F*^2^ > 2σ(*F*^2^)] = 0.059	H-atom parameters constrained
*wR*(*F*^2^) = 0.158	*w* = 1/[σ^2^(*F*_o_^2^) + (0.0582*P*)^2^ + 2.927*P*] where *P* = (*F*_o_^2^ + 2*F*_c_^2^)/3
*S* = 1.04	(Δ/σ)_max_ = 0.001
8451 reflections	Δρ_max_ = 0.56 e Å^−^^3^
307 parameters	Δρ_min_ = −0.59 e Å^−^^3^
164 restraints	Absolute structure: Flack (1983), 4226 Friedel pairs
Primary atom site location: structure-invariant direct methods	Absolute structure parameter: 0.38 (17)

### Special details

**Table:**

Geometry. All e.s.d.'s (except the e.s.d. in the dihedral angle between two l.s. planes) are estimated using the full covariance matrix. The cell e.s.d.'s are taken into account individually in the estimation of e.s.d.'s in distances, angles and torsion angles; correlations between e.s.d.'s in cell parameters are only used when they are defined by crystal symmetry. An approximate (isotropic) treatment of cell e.s.d.'s is used for estimating e.s.d.'s involving l.s. planes.
Refinement. Refined as a 2-component inversion twin (here a polar twin) with a component ratio of 0.38/0.62. The final refinement restrained the four independent molecules to similar geometry using the SAME command of *SHELXL*, and EADP constraints were applied to equivalent atom pairs related by pseudosymmetry. Some residual electron density appeared to arise from partial disorder of the ethylene groups of two of the molecules over two sites (0.78:0.22) so the C atoms of the minor component were included with fixed isotropic thermal parameters.

### Fractional atomic coordinates and isotropic or equivalent isotropic displacement parameters (Å^2^)

**Table:**

	*x*	*y*	*z*	*U*_iso_*/*U*_eq_	Occ. (<1)
C1	0.1793 (6)	0.69065 (18)	0.5906 (7)	0.0257 (6)	
H1A	0.2429	0.6694	0.5886	0.031*	
H1B	0.1907	0.7095	0.5168	0.031*	
C2	0.0503 (6)	0.67256 (17)	0.5692 (6)	0.0295 (6)	
H2A	−0.0133	0.6940	0.5615	0.035*	
H2B	0.0420	0.6572	0.4843	0.035*	
C3	0.0387 (5)	0.66860 (16)	0.8026 (6)	0.0299 (6)	
H3A	0.0211	0.6505	0.8767	0.036*	
H3B	−0.0244	0.6901	0.7973	0.036*	
C4	0.1672 (5)	0.68631 (15)	0.8331 (6)	0.0245 (6)	
H4A	0.1701	0.7025	0.9161	0.029*	
H4B	0.2300	0.6648	0.8478	0.029*	
C5	0.2610 (4)	0.74577 (14)	0.7460 (5)	0.0216 (5)	
C6	0.3614 (4)	0.80530 (13)	0.6609 (5)	0.0241 (6)	
H6A	0.3323	0.8190	0.7397	0.029*	
H6B	0.3423	0.8225	0.5810	0.029*	
C7	0.4988 (4)	0.79800 (13)	0.6835 (6)	0.0317 (7)	
H7A	0.5272	0.7829	0.6070	0.038*	
H7B	0.5187	0.7822	0.7666	0.038*	
N1	0.1972 (4)	0.71155 (12)	0.7195 (5)	0.0201 (5)	
N2	0.2967 (4)	0.76644 (11)	0.6398 (4)	0.0308 (5)	
H2C	0.2820 (5)	0.7572 (2)	0.5612 (18)	0.030*	
O1	0.0284 (3)	0.64694 (11)	0.6785 (4)	0.0297 (4)	
O2	0.2846 (3)	0.75889 (10)	0.8630 (4)	0.0292 (4)	
Cl1	0.57778 (12)	0.84633 (4)	0.69906 (14)	0.0372 (2)	
C8	0.6779 (6)	0.56216 (18)	1.0240 (7)	0.0257 (6)	
H8A	0.6654	0.5439	1.0993	0.031*	
H8B	0.6127	0.5830	1.0210	0.031*	
C9	0.8058 (6)	0.58091 (17)	1.0459 (6)	0.0295 (6)	
H9A	0.8108	0.5971	1.1291	0.035*	
H9B	0.8695	0.5596	1.0592	0.035*	
C10	0.8262 (5)	0.58296 (16)	0.8153 (6)	0.0299 (6)	
H10A	0.8900	0.5616	0.8237	0.036*	
H10B	0.8445	0.6005	0.7401	0.036*	
C11	0.6986 (5)	0.56463 (15)	0.7835 (6)	0.0245 (6)	
H11A	0.6352	0.5859	0.7661	0.029*	
H11B	0.6979	0.5481	0.7016	0.029*	
C12	0.5946 (4)	0.50722 (14)	0.8711 (5)	0.0216 (5)	
C13	0.4970 (5)	0.44776 (15)	0.9575 (6)	0.0241 (6)	0.778 (4)
H13A	0.5150	0.4313	1.0393	0.029*	
H13B	0.5310	0.4340	0.8815	0.029*	
C14	0.3576 (5)	0.45371 (16)	0.9277 (7)	0.0317 (7)	0.778 (4)
H14A	0.3393	0.4683	0.8420	0.038*	
H14B	0.3249	0.4694	1.0004	0.038*	
N3	0.6675 (4)	0.54001 (12)	0.8967 (5)	0.0201 (5)	
N4	0.5548 (4)	0.48731 (11)	0.9778 (4)	0.0308 (5)	
H4C	0.5635 (4)	0.4978 (3)	1.0565 (18)	0.030*	
O3	0.8346 (3)	0.60560 (10)	0.9370 (4)	0.0297 (4)	
O4	0.5687 (3)	0.49474 (11)	0.7551 (4)	0.0292 (4)	
Cl2	0.28368 (12)	0.40510 (4)	0.91603 (15)	0.0372 (2)	
C15	0.1664 (6)	0.81124 (18)	0.0959 (7)	0.0268 (6)	
H15A	0.1830	0.7940	0.0194	0.032*	
H15B	0.2269	0.8335	0.1003	0.032*	
C16	0.0353 (6)	0.82743 (18)	0.0734 (6)	0.0317 (6)	
H16A	0.0273	0.8440	−0.0089	0.038*	
H16B	−0.0244	0.8050	0.0592	0.038*	
C17	0.0137 (5)	0.82715 (16)	0.3030 (6)	0.0283 (6)	
H17A	−0.0465	0.8048	0.2915	0.034*	
H17B	−0.0088	0.8437	0.3790	0.034*	
C18	0.1445 (5)	0.81076 (16)	0.3359 (6)	0.0245 (6)	
H18A	0.2035	0.8330	0.3580	0.029*	
H18B	0.1461	0.7932	0.4156	0.029*	
C19	0.2757 (4)	0.76025 (14)	0.2480 (5)	0.0220 (5)	
C20	0.4416 (4)	0.71961 (12)	0.1643 (5)	0.0251 (6)	
H20A	0.4928	0.7283	0.2470	0.030*	
H20B	0.4929	0.7226	0.0879	0.030*	
C21	0.4066 (4)	0.67709 (12)	0.1778 (6)	0.0270 (6)	
H21A	0.3554	0.6680	0.0956	0.032*	
H21B	0.3571	0.6736	0.2554	0.032*	
N5	0.1841 (4)	0.78819 (12)	0.2215 (5)	0.0199 (4)	
N6	0.3293 (4)	0.74516 (11)	0.1419 (4)	0.0298 (5)	
H6C	0.2982 (8)	0.75040 (16)	0.0619 (18)	0.030*	
O5	0.0041 (4)	0.85058 (10)	0.1835 (4)	0.0321 (4)	
O6	0.3081 (3)	0.74858 (11)	0.3645 (4)	0.0281 (4)	
Cl3	0.55040 (11)	0.64849 (4)	0.20263 (14)	0.03441 (18)	
C22	0.6776 (6)	0.94101 (19)	0.5227 (7)	0.0268 (6)	
H22A	0.6175	0.9186	0.5190	0.032*	
H22B	0.6605	0.9586	0.5981	0.032*	
C23	0.8101 (6)	0.92532 (18)	0.5449 (6)	0.0317 (6)	
H23A	0.8690	0.9480	0.5557	0.038*	
H23B	0.8199	0.9095	0.6289	0.038*	
C24	0.8292 (5)	0.92350 (16)	0.3134 (6)	0.0283 (6)	
H24A	0.8502	0.9064	0.2382	0.034*	
H24B	0.8895	0.9459	0.3216	0.034*	
C25	0.6977 (5)	0.93984 (16)	0.2819 (6)	0.0245 (6)	
H25A	0.6942	0.9568	0.2006	0.029*	
H25B	0.6380	0.9176	0.2637	0.029*	
C26	0.5745 (5)	0.99248 (14)	0.3691 (5)	0.0220 (5)	
C27	0.4059 (5)	1.03184 (15)	0.4504 (6)	0.0251 (6)	0.778 (4)
H27A	0.3535	1.0278	0.5254	0.030*	
H27B	0.3574	1.0232	0.3660	0.030*	
C28	0.4398 (5)	1.07526 (15)	0.4409 (6)	0.0270 (6)	0.778 (4)
H28A	0.4897	1.0796	0.3641	0.032*	
H28B	0.4900	1.0839	0.5242	0.032*	
N7	0.6630 (4)	0.96323 (12)	0.3958 (5)	0.0199 (4)	
N8	0.5208 (4)	1.00824 (11)	0.4736 (4)	0.0298 (5)	
H8C	0.5529 (8)	1.00457 (14)	0.5510 (18)	0.030*	
O7	0.8412 (4)	0.90094 (10)	0.4351 (4)	0.0321 (4)	
O8	0.5435 (3)	1.00387 (11)	0.2531 (4)	0.0281 (4)	
Cl4	0.29550 (11)	1.10319 (3)	0.41732 (14)	0.03441 (18)	
C13A	0.421 (2)	0.4712 (5)	0.954 (2)	0.030*	0.222 (4)
C14A	0.442 (2)	0.4253 (5)	0.939 (2)	0.030*	0.222 (4)
C27A	0.4955 (18)	1.0541 (6)	0.460 (2)	0.030*	0.222 (4)
C28A	0.3506 (17)	1.0513 (6)	0.427 (2)	0.030*	0.222 (4)

### Atomic displacement parameters (Å^2^)

**Table:**

	*U*^11^	*U*^22^	*U*^33^	*U*^12^	*U*^13^	*U*^23^
C1	0.0285 (14)	0.0265 (13)	0.0224 (14)	−0.0047 (11)	0.0047 (11)	−0.0045 (11)
C2	0.0326 (15)	0.0302 (14)	0.0251 (15)	−0.0090 (12)	0.0001 (11)	0.0000 (11)
C3	0.0297 (15)	0.0330 (13)	0.0280 (15)	−0.0047 (12)	0.0074 (12)	0.0043 (11)
C4	0.0281 (15)	0.0246 (12)	0.0203 (13)	−0.0047 (11)	0.0006 (11)	0.0037 (10)
C5	0.0194 (12)	0.0249 (12)	0.0204 (13)	0.0015 (10)	0.0019 (10)	0.0010 (10)
C6	0.0223 (14)	0.0251 (14)	0.0244 (14)	0.0013 (12)	0.0005 (11)	0.0010 (11)
C7	0.0235 (15)	0.0299 (14)	0.0420 (18)	0.0007 (12)	0.0056 (13)	0.0004 (13)
N1	0.0217 (12)	0.0223 (10)	0.0170 (11)	−0.0050 (9)	0.0049 (9)	−0.0001 (8)
N2	0.0403 (13)	0.0351 (12)	0.0168 (11)	−0.0182 (10)	0.0019 (9)	−0.0016 (9)
O1	0.0318 (11)	0.0288 (9)	0.0282 (11)	−0.0122 (8)	0.0021 (8)	0.0027 (8)
O2	0.0414 (11)	0.0290 (9)	0.0172 (9)	−0.0094 (8)	0.0037 (8)	−0.0030 (7)
Cl1	0.0332 (4)	0.0381 (4)	0.0398 (4)	−0.0168 (3)	0.0012 (3)	−0.0013 (3)
C8	0.0285 (14)	0.0265 (13)	0.0224 (14)	−0.0047 (11)	0.0047 (11)	−0.0045 (11)
C9	0.0326 (15)	0.0302 (14)	0.0251 (15)	−0.0090 (12)	0.0001 (11)	0.0000 (11)
C10	0.0297 (15)	0.0330 (13)	0.0280 (15)	−0.0047 (12)	0.0074 (12)	0.0043 (11)
C11	0.0281 (15)	0.0246 (12)	0.0203 (13)	−0.0047 (11)	0.0006 (11)	0.0037 (10)
C12	0.0194 (12)	0.0249 (12)	0.0204 (13)	0.0015 (10)	0.0019 (10)	0.0010 (10)
C13	0.0223 (14)	0.0251 (14)	0.0244 (14)	0.0013 (12)	0.0005 (11)	0.0010 (11)
C14	0.0235 (15)	0.0299 (14)	0.0420 (18)	0.0007 (12)	0.0056 (13)	0.0004 (13)
N3	0.0217 (12)	0.0223 (10)	0.0170 (11)	−0.0050 (9)	0.0049 (9)	−0.0001 (8)
N4	0.0403 (13)	0.0351 (12)	0.0168 (11)	−0.0182 (10)	0.0019 (9)	−0.0016 (9)
O3	0.0318 (11)	0.0288 (9)	0.0282 (11)	−0.0122 (8)	0.0021 (8)	0.0027 (8)
O4	0.0414 (11)	0.0290 (9)	0.0172 (9)	−0.0094 (8)	0.0037 (8)	−0.0030 (7)
Cl2	0.0332 (4)	0.0381 (4)	0.0398 (4)	−0.0168 (3)	0.0012 (3)	−0.0013 (3)
C15	0.0322 (16)	0.0287 (13)	0.0202 (14)	0.0083 (12)	0.0063 (11)	0.0035 (10)
C16	0.0370 (16)	0.0325 (14)	0.0244 (15)	0.0131 (12)	−0.0034 (12)	−0.0021 (11)
C17	0.0247 (14)	0.0296 (13)	0.0310 (15)	0.0063 (11)	0.0056 (11)	−0.0024 (11)
C18	0.0249 (14)	0.0261 (12)	0.0227 (14)	0.0040 (11)	0.0042 (11)	−0.0027 (10)
C19	0.0245 (13)	0.0234 (12)	0.0185 (13)	−0.0012 (10)	0.0043 (10)	−0.0012 (10)
C20	0.0209 (15)	0.0286 (13)	0.0271 (15)	−0.0016 (12)	0.0082 (11)	−0.0033 (11)
C21	0.0211 (15)	0.0274 (14)	0.0322 (16)	−0.0013 (12)	0.0005 (12)	−0.0019 (12)
N5	0.0233 (11)	0.0192 (9)	0.0175 (11)	0.0023 (8)	0.0028 (8)	0.0001 (8)
N6	0.0396 (13)	0.0344 (11)	0.0156 (11)	0.0191 (10)	0.0035 (9)	0.0012 (9)
O5	0.0360 (11)	0.0303 (9)	0.0293 (11)	0.0126 (8)	0.0001 (8)	−0.0031 (8)
O6	0.0343 (10)	0.0338 (9)	0.0163 (9)	0.0116 (8)	0.0031 (7)	0.0021 (7)
Cl3	0.0318 (4)	0.0353 (3)	0.0355 (4)	0.0150 (3)	0.0002 (3)	−0.0016 (3)
C22	0.0322 (16)	0.0287 (13)	0.0202 (14)	0.0083 (12)	0.0063 (11)	0.0035 (10)
C23	0.0370 (16)	0.0325 (14)	0.0244 (15)	0.0131 (12)	−0.0034 (12)	−0.0021 (11)
C24	0.0247 (14)	0.0296 (13)	0.0310 (15)	0.0063 (11)	0.0056 (11)	−0.0024 (11)
C25	0.0249 (14)	0.0261 (12)	0.0227 (14)	0.0040 (11)	0.0042 (11)	−0.0027 (10)
C26	0.0245 (13)	0.0234 (12)	0.0185 (13)	−0.0012 (10)	0.0043 (10)	−0.0012 (10)
C27	0.0209 (15)	0.0286 (13)	0.0271 (15)	−0.0016 (12)	0.0082 (11)	−0.0033 (11)
C28	0.0211 (15)	0.0274 (14)	0.0322 (16)	−0.0013 (12)	0.0005 (12)	−0.0019 (12)
N7	0.0233 (11)	0.0192 (9)	0.0175 (11)	0.0023 (8)	0.0028 (8)	0.0001 (8)
N8	0.0396 (13)	0.0344 (11)	0.0156 (11)	0.0191 (10)	0.0035 (9)	0.0012 (9)
O7	0.0360 (11)	0.0303 (9)	0.0293 (11)	0.0126 (8)	0.0001 (8)	−0.0031 (8)
O8	0.0343 (10)	0.0338 (9)	0.0163 (9)	0.0116 (8)	0.0031 (7)	0.0021 (7)
Cl4	0.0318 (4)	0.0353 (3)	0.0355 (4)	0.0150 (3)	0.0002 (3)	−0.0016 (3)

### Geometric parameters (Å, º)

**Table:**

C1—N1	1.463 (6)	C15—C16	1.506 (7)
C1—C2	1.509 (7)	C16—O5	1.415 (6)
C2—O1	1.429 (6)	C17—O5	1.426 (6)
C3—O1	1.433 (6)	C17—C18	1.514 (6)
C3—C4	1.506 (6)	C18—N5	1.470 (6)
C4—N1	1.478 (6)	C19—O6	1.244 (6)
C5—O2	1.252 (6)	C19—N6	1.354 (6)
C5—N1	1.352 (6)	C19—N5	1.367 (6)
C5—N2	1.355 (6)	C20—N6	1.480 (5)
C6—N2	1.485 (5)	C20—C21	1.487 (5)
C6—C7	1.491 (5)	C21—Cl3	1.815 (5)
C7—Cl1	1.832 (4)	C22—N7	1.467 (6)
C8—N3	1.469 (6)	C22—C23	1.513 (7)
C8—C9	1.507 (7)	C23—O7	1.435 (6)
C9—O3	1.427 (6)	C24—O7	1.428 (6)
C10—O3	1.430 (6)	C24—C25	1.519 (6)
C10—C11	1.507 (6)	C25—N7	1.462 (6)
C11—N3	1.466 (6)	C26—O8	1.235 (5)
C12—O4	1.239 (6)	C26—N8	1.351 (6)
C12—N3	1.362 (6)	C26—N7	1.375 (6)
C12—N4	1.362 (6)	C27—N8	1.466 (6)
C13—N4	1.473 (6)	C27—C28	1.509 (7)
C13—C14	1.510 (7)	C28—Cl4	1.807 (5)
C14—Cl2	1.815 (5)	C13A—C14A	1.568 (16)
C15—N5	1.471 (6)	C27A—C28A	1.561 (17)
			
N1—C1—C2	110.9 (5)	N5—C15—C16	110.9 (5)
O1—C2—C1	110.7 (5)	O5—C16—C15	111.9 (5)
O1—C3—C4	111.5 (5)	O5—C17—C18	111.8 (5)
N1—C4—C3	109.7 (5)	N5—C18—C17	110.7 (5)
O2—C5—N1	122.3 (5)	O6—C19—N6	120.9 (4)
O2—C5—N2	120.3 (4)	O6—C19—N5	121.8 (5)
N1—C5—N2	117.4 (5)	N6—C19—N5	117.3 (5)
N2—C6—C7	108.6 (4)	N6—C20—C21	111.2 (4)
C6—C7—Cl1	108.0 (3)	C20—C21—Cl3	107.6 (3)
C5—N1—C1	126.9 (5)	C19—N5—C18	117.6 (5)
C5—N1—C4	118.9 (5)	C19—N5—C15	123.8 (5)
C1—N1—C4	112.4 (4)	C18—N5—C15	111.6 (4)
C5—N2—C6	120.3 (4)	C19—N6—C20	120.1 (4)
C2—O1—C3	110.6 (4)	C16—O5—C17	110.2 (4)
N3—C8—C9	109.1 (5)	N7—C22—C23	108.8 (5)
O3—C9—C8	113.4 (5)	O7—C23—C22	112.0 (5)
O3—C10—C11	112.1 (5)	O7—C24—C25	111.8 (5)
N3—C11—C10	109.9 (5)	N7—C25—C24	109.7 (5)
O4—C12—N3	121.4 (5)	O8—C26—N8	120.5 (4)
O4—C12—N4	120.5 (4)	O8—C26—N7	121.4 (5)
N3—C12—N4	118.1 (5)	N8—C26—N7	118.0 (5)
N4—C13—C14	107.7 (4)	N8—C27—C28	109.2 (4)
C13—C14—Cl2	108.2 (4)	C27—C28—Cl4	107.5 (4)
C12—N3—C11	118.9 (5)	C26—N7—C25	117.3 (5)
C12—N3—C8	124.4 (5)	C26—N7—C22	123.0 (5)
C11—N3—C8	112.3 (4)	C25—N7—C22	112.7 (4)
C12—N4—C13	119.8 (4)	C26—N8—C27	120.5 (4)
C9—O3—C10	109.8 (4)	C24—O7—C23	109.9 (4)

### Hydrogen-bond geometry (Å, º)

**Table:**

*D*—H···*A*	*D*—H	H···*A*	*D*···*A*	*D*—H···*A*
N2—H2*C*···O6	0.85	2.03	2.831 (6)	157
N4—H4*C*···O4^i^	0.86	2.00	2.826 (6)	162
N6—H6*C*···O2^ii^	0.85	2.00	2.819 (6)	161
N8—H8*C*···O8^iii^	0.82	2.05	2.809 (6)	153

Symmetry codes: (i) *x*, −*y*+1, *z*+1/2; (ii) *x*, *y*, *z*−1; (iii) *x*, −*y*+2, *z*+1/2.
